# “It’s about maintaining a calm and reassuring presence”: a qualitative study about ambulance clinicians’ experiences of caring for patients with breathlessness

**DOI:** 10.1186/s12873-025-01437-z

**Published:** 2025-12-05

**Authors:** Wivica Kauppi, Hanna Maurin Söderholm, Magnus Andersson Hagiwara

**Affiliations:** 1 PreHospen- Centre for Prehospital Research, Faculty of Caring Science, Work Life and Social Welfare, University of Borås, Borås, Sweden; 2 Faculty of Caring Science, Work Life and Social Welfare, University of Borås, Borås, Sweden; 3 PICTA- Prehospital Innovation Arena, Lindholmen Science Park, Gothenburg, Sweden

**Keywords:** Breathlessness, Dyspnoea, Caring, Ambulance clinician, Experience, Assessment, Ambulance services, Pre-hospital

## Abstract

**Background:**

Despite breathlessness (dyspnoea) being a common and serious symptom that often necessitates ambulance care, there is still a limited understanding of how ambulance clinicians (ACs) experience and manage these situations in the pre-hospital setting. Given the potential severity and complexity of breathlessness, as well as its impact on patient outcomes, a deeper insight into ACs’ perspectives is essential. This study aimed to explore ACs experiences of caring for patients with breathlessness within ambulance services.

**Methods:**

An inductive qualitative design was employed. Data were collected through dyadic interviews with 16 ACs from two ambulance organizations in southwestern Sweden during two days in May and June 2021. The data were analyzed using qualitative content analysis.

**Results:**

The ACs experienced several challenges in caring for patients with breathlessness, which is captured in three generic categories: navigating patients’ experiences during breathlessness, navigating relatives and their involvement, and navigating one’s own capability in care. ACs highlighted challenges of identifying and addressing patients’ individual needs, emphasizing the crucial role of trust-building. While ACs relied on their clinical expertise, assessing a patient’s condition remained challenging in certain situations. Furthermore, their ability to manage stress and emotional demands were essential for providing quality care.

**Conclusions:**

This study underscores the challenges of caring for patients with breathlessness, with ACs employing strategies to manage stress and establish a safe environment. Clinical experience and ongoing training and education are strategies to strengthen clinical reasoning and decision-making. Pairing less experienced ACs with seasoned colleagues could also improve care quality and support the development of professional competence.

**Supplementary Information:**

The online version contains supplementary material available at 10.1186/s12873-025-01437-z.

## Background

Breathlessness (dyspnoea) is a common and distressing symptom and a frequent reason why patients require pre-hospital care provided by ambulance clinicians (ACs) [1, 2]. According to the American Thoracic Society, breathlessness is described as a subjective sensation of breathing discomfort, comprising qualitatively distinct experiences that may vary in intensity. It can present in different forms, such as shortness of breath, air hunger, or chest tightness [3]. Breathlessness is associated with a wide range of underlying conditions. These include temporary or chronic conditions such as anxiety, chronic obstructive pulmonary disease, or asthma. Breathlessness may also signal severe and potentially life-threatening conditions such as acute heart failure, pulmonary embolism, or acute myocardial infarction [4, 6]. Several of these are linked to high mortality rates [7]. The presentation of breathlessness can vary widely, requiring ACs to be adept at recognizing and managing its different underlying causes [8, 9]. ACs operate independently in varied and unpredictable environments, providing personalized care from a holistic perspective addressing physical, psychological, existential, and social needs [10]. *Existential needs* refer to patients’ concerns related to uncertainty, vulnerability, and the threat to life. Although partly overlapping with psychological needs, the concept encompasses a broader dimension. These needs become particularly significant in acute and critical situations, where symptoms and uncertainty can intensify feelings of vulnerability and loss of control [11, 12]. The experience of breathlessness varies greatly between individuals [13], necessitating a broad range of competencies in ACs including e.g. medical, psychological and existential knowledge, leadership, communication and technical skills [14, 15]. Caring for patients with breathlessness in ambulance services differs significantly from hospital care, where clinicians have access to a multidisciplinary team, advanced equipment, and a wider range of treatment options. These additional resources allow for more comprehensive management than is possible in the resource-limited ambulance setting [16, 18]. Based on their initial assessment of the patient’s condition, ACs determine the urgency of patient’s condition and appropriate level of care, whether hospital transport is necessary for further evaluation and treatment [19, 20]. Delays in accurately identifying the primary cause of the patient’s complaint can result in increased morbidity and prolonged hospital stays [21]. From the patients’ perspective, previous studies have shown that the emotional state displayed by ACs, combined with their confidence in their caregiving abilities, enhances patients’ sense of safety and security. This contributes to better care experiences and greater trust in the care provided [22, 23]. Similarly, patients assume that ACs possess the necessary competence to care for those experiencing breathlessness [24]. However, ACs’ experiences of caring for patients with breathlessness remain limited. Existing studies mainly explore the general experience of providing care within ambulance services [25, 26]. Research has also focused on patients with specific conditions, such as myocardial infarction, stroke [27, 28], or chest pain [29], in which breathlessness may occur but is not the primary complaint. Breathlessness as a standalone symptom presents unique challenges for ACs, given the complex interplay of patients’ medical and existential needs in these situations [1, 7, 24, 30]. Understanding ACs’ perspectives and gaining contextual insights on caring for patients with breathlessness in the ambulance service may have a direct impact on the quality of care. Such insights can contribute to improvements in nursing practice and enhance patient outcomes. Therefore, the aim of this study was to explore ACs’ experiences of caring for patients with breathlessness in the context of ambulance services. By focusing on this specific symptom, the study seeks to fill a gap in the existing literature and provide valuable insights that can inform and improve pre-hospital care practices. In this study, the term pre-hospital care is used broadly to refer to all out-of-hospital care conducted by ACs.

## Methods

### Study design

This study is the first of two sub-studies exploring ACs experiences of caring for patients with breathlessness in ambulance services. A qualitative design with an inductive approach was employed to explore these experiences, allowing for in-depth insights into the participants’ perspectives [31, 32]. This design included a simulated scenario to create a realistic care environment, which served as the basis for the subsequent dyadic interviews. The simulation was used solely to stimulate reflection during the subsequent interviews, rather than to observe or analyze participants’ behavior. It provided a shared scenario-based starting point that helped participants articulate and elaborate on their reflections, drawing on both the simulated event and their prior experiences of caring for patients with breathlessness. By combining simulation with dyadic interviews, the study aimed to gain a comprehensive understanding of participants’ experiences in providing care for patients with breathlessness [33, 34]. The data were collected over two consecutive days at the turn of May and June 2021. The COREQ (Consolidated criteria for Reporting Qualitative research) checklist was used to ensure comprehensive and transparent reporting of the study [35] (see Additional file 1).

### Setting and participants

The study was conducted in two ambulance organizations encompassing five ambulance stations in the southwestern region of Sweden. These organizations serve a diverse patient population across urban, suburban and rural areas. In Sweden, ambulances staffed by ACs must include at least one registered nurse (RN), which is a fundamental requirement for the provision of ambulance services [36, 37]. Ambulance crews typically consist of RNs, most commonly specialized in pre-hospital emergency care, though occasionally with backgrounds in areas such as intensive care or anesthesia. Additionally, the team composition often includes ambulance technicians with backgrounds as assistant nurses.

Participants were initially selected using purposive sampling. Subsequently, existing participants and authors of this study assisted in recruiting additional participants using the snowball sampling method, as the target group was considered difficult to reach [38]. One possible reason for this was that participation required ACs to be available on predetermined data collection days and willing to complete the simulations and interviews during their time off. The inclusion criteria required participants to be RNs working in ambulance services with additional specialist training in either ambulance care, intensive care, or anesthesia. Efforts were made to ensure variation in age, gender, and years of clinical experience in ambulance services, along with representation from both urban, suburban and rural settings. Additionally, representation from various ambulance stations within the target organizations was considered desirable.

After obtaining consent from chief executive officers, participants were recruited through multiple channels, including direct messages via an internal platform, social media posts, and targeted invitations. Participants who agreed to take part in the study, which included a simulation followed by an interview, were given the opportunity to express their preferences regarding which of the two days they wished to participate. In addition, when participants expressed preferences for collaboration partners during the simulation and interview, these preferences were considered. No participants withdrew from the study. In total, 16 participants were included in the study, consisting of 11 men and 5 women, aged 27 to 52 years. Included participants had specialized training in either ambulance care (*n* = 12), intensive care (*n* = 1), or anesthesia care (*n* = 1). Two participants held dual qualifications: one in both anesthesia- and intensive care (*n* = 1), and another in both ambulance- and intensive care (*n* = 1). Participants’ clinical experience in the field of ambulance services ranged from 1 to 22 years (see Table 1), with median of 6 years. The included participants represented care provided in urban, suburban, and rural settings. They also represented various ambulance stations, which offered a comprehensive range of perspectives within the field of ambulance care.

**Table 1:** Participants characteristics

	*n* = 16
**Gender**	
Male	11
Female	5
**Age (years)**	
25–34	5
35–44	5
45–54	6
**Clinical experience in ambulance services (years)**	
0–3 years	3
4–6 years	6
7–10 years	2
11–15 years	2
16–22 years	3
**Registered nurse with area of specialization**	
Ambulance care	12
Intensive care	1
Anesthesia care	1
Ambulance- and Intensive care	1
Anesthesia- and Intensive care	1

### The simulation

Before the interviews, participants engaged in a simulation, working in pairs as ambulance crew members. Each pair participated in the same simulation scenario, with a total of eight simulations conducted across all participants. They responded to a dispatcher alarm about a breathless patient and provided care in a fully equipped ambulance, replicating a real-life care scenario. The scenario involved a live actor portraying a breathless patient, creating a realistic environment that evoked genuine emotional and cognitive responses. The same actor portrayed the patient in all scenarios, ensuring that all participants engaged in the same scenario. The actor followed a pre-defined script to maintain consistent symptoms and behavior. Such immersion was essential for generating rich qualitative data in the subsequent interviews as participants reflected on their experiences [39]. Additionally, instructions, environment, and equipment were identical for all participants. Although participants were not informed about the specific scenario in advance, they had provided informed consent and were fully briefed on the study’s overall purpose, procedures, and the use of simulation followed by interviews. Only the specific scenario details were withheld to support natural responses, and participants were simply instructed to work as usual to maintain authentic behavior [40]. Their expertise and experience were of particular interest, as it was not feasible to observe and discuss their care for patients with breathlessness in real-life care situations. During the scenarios, a live-stream was used solely to allow facilitators to follow the scenario remotely without entering the simulation site, or disrupting the participants.

### Data collection

Data were collected through eight dyadic interviews, meaning that two participants are interviewed together [41]. Following the simulations, semi-structured interviews were conducted face-to-face near the simulation site, allowing for the collection of immediate and contextually rich insights into participants’ experiences. Participants reflected on the simulation, considering its relevance to their understanding of care for patients with breathlessness. This process also prompted them to connect with their prior experiences in ambulance services. The questions were partly designed, inspired by previous studies on patients’ experiences of breathlessness prior to and during ambulance care [24, 30], but were intentionally kept open to allow for an inductive exploration of the participants’ own experiences. Each interview began with the question: “*How did you experience caring for this patient who was struggling with her breathing?”* (see Table 2 for additional questions). Follow-up questions aimed to delve deeper into participants’ experiences, such as *“What do you mean by…?”*,* “Can you describe it more?”*, and *“Can you give an example of*…?”. Each interview lasted between 23 and 47 min, with a median duration of 39 min. The interviews were conducted by the first author (n = 5), the second author (n = 1), and an external researcher with interviewing experience (n = 2). This approach was necessary due to the tight schedule between simulations and the first author’s personal connection with participants in three of the ambulance crews. Due to practical constraints, data collection was not conducted with the aim of achieving data saturation. Instead, the study was based on those participants who expressed interest in taking part in the simulation followed by an interview. However, the interviews conducted generated rich material that was sufficient to address the study aim. All interviews were audio recorded and transcribed verbatim. After transcription, the audio recordings were securely stored with access restricted to the authors of the study.

**Table 2:** Overview of interview questions used in the study

No	Interview Question	
1	How did you experience caring for this patient who was struggling with her breathing?	
2	What previous experiences do you have of caring for patients with breathlessness?	
3	Do you think patients with breathlessness wait a long time before seeking help?	
4	Can you recognize death anxiety in these patients?	
5	How do you approach a patient who feels they are close to death?	
6	What do you think a patient with breathlessness needs?	
7	What do you think is the most important aspect of caring for a patient with breathlessness?	
8	How do you usually calm a patient with breathlessness?	
9	Is there anything else you would like to add that you think is important in caring for patients with breathlessness?	

### Data analysis

Data were analyzed using the inductive qualitative content analysis method as described by Elo and Kyngäs (2008) [31]. This process involved moving back and forth between the complete dataset and its individual parts in order to develop a comprehensive understanding of the data. The analysis focused on the manifest content, meaning that interpretations remained close to the text. The analysis was conducted in three phases: preparation, organization, and reporting. In the preparation phase, the interviews were read and reread to gain a deeper understanding of the overall content. Meaning units were then identified which are segments of the text that provide relevant information about the aim being studied. During the organizational phase, open coding was performed. This involved organizing the material by content and labelling it accordingly. The text was abstracted by asking questions such as “What does this mean?” and “How can I be sure of that?” The aim was to identify related meanings that aligned with the study’s aim. These meanings were then examined across the interviews to ensure consistency. This process led to the development of subcategories, which were linked by their common meanings to form generic categories. Ultimately, three generic categories and ten subcategories emerged from the data.

### Rigour

To ensure credibility and trustworthiness [32], all authors reflected on their pre-understandings and the first and last authors carefully conducted the analysis process (see Table 3). In addition, an external researcher critically reviewed the coding framework and the emerging categories, which further enhanced the dependability of the results. Constructive feedback from external reviewers was incorporated during the revision process, contributing to greater transparency and clarity in the final manuscript. Quotes from the interviews were included to enhance confirmability.

**Table 3:** Examples of analysis process

Raw data/Quote	Code	Subcategory	Generic category
*“The goal is to relieve the situation. If you reduce their anxiety*,* you improve their breathing”*	The necessity of anxiety relief	Building trust	Navigating patients’ experiences during breathlessness
*“.you’re in a situation where it’s a matter of life and death.I feel that they sort of just surrender themselves as well”*	Dependent on others	Building trust	Navigating patients’ experiences during breathlessness
*“If you describe things to the patient.then you create some kind of control for the patient”*	The necessity of information	Building trust	Navigating patients’ experiences during breathlessness

### Ethical considerations

This study was approved by the Swedish Ethical Review Authority (Dnr 2019–03283), and adhered to the ethical principles outlined in the Helsinki Declaration [42]. Participants were provided with both written and verbal information about the study’s purpose, design, and their right to withdraw at any time without consequences. Informed consent was obtained from all participants. All data collection complied with the General Data Protection Regulation (GDPR) according to the university’s regulations.

## Results

**Fig. 1:**
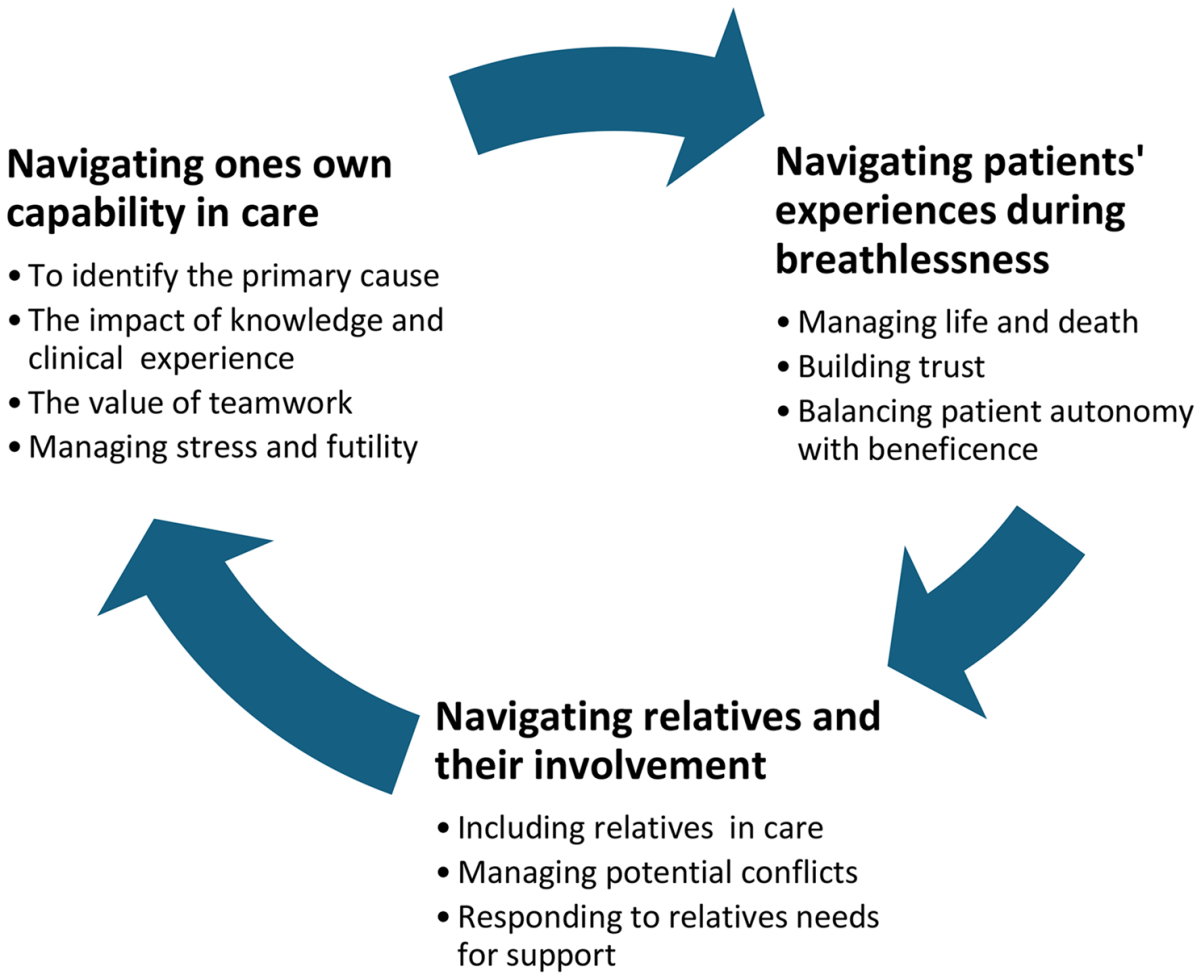
Description of generic categories and subcategories

The following generic categories and subcategories elucidate the structure of ACs experiences of caring for patients with breathlessness (Fig. 1).

### Navigating patients’ experiences during breathlessness

ACs observe various expressions of death anxiety in patients with breathlessness, ranging from panic to withdrawal. They prioritize creating a calm environment to help alleviate this anxiety and focus on building trust through clear and transparent communication. Recognizing the importance of addressing both physical and existential needs, ACs understand that symptom relief can help stabilize emotions. They emphasize the value of their steady presence and effective teamwork in enhancing patients’ sense of security, reassuring them that they will receive the necessary care. Meeting the patient’s intertwined existential and medical needs is described as essential for fostering a sense of control, thereby reducing anxiety.

### Managing life and death

ACs observe that patients experiencing breathlessness exhibit death anxiety in various ways. Some visibly panic, hyperventilating, staring wide-eyed, or displaying restlessness, while others appear to struggle intensely, using their whole body to draw in air.

Yes, there’s a lot of anxiety in the room. You can see they’re just struggling, that patients feels like ‘this is the end because I can’t breathe’. So, they’re using their whole body to try to get air in (Int1/Part1)

While some patients express their death anxiety through shouting or physical agitation as described by ACs, others manage their breathlessness with surprising calmness, showing less overt panic despite their distress. However, a concerning pattern emerges from the observations of ACs when patients eventually fall silent, shutting down as their anxiety escalates. In more critical situations, patients may become unresponsive. This progression highlights the complex relationship between patients’ feelings and physical condition during these critical moments.

When they express ‘I can’t breathe’ and ‘I’m going to die’.that’s when there’s an explicit fear of death. But my experience tells me that it doesn’t stop there. After that stage, there’s another step where they go completely silent and probably become entirely introverted. There’s likely still a fear of death at that point, but it doesn’t come out verbally. Instead, it’s like they shut down entirely (Int3/Part2)

### Building trust

In caring for patients with breathlessness, ACs prioritize creating a calm and secure environment, considering this approach central to alleviating patient anxiety. They focus on building trust and communicating clearly to reduce stress in order to help patients feel a sense of control despite their distress. ACs recognize that patients often experience significant anxiety alongside breathlessness, which can further worsen their condition and should be avoided.

The goal is to relieve the situation. If you reduce their anxiety, you improve their breathing. And there’s the medical side of things, like reducing oxygen consumption and similar issues (Int4/Part2)

In critical situations where time for existential support is limited, ACs focus on prioritizing medical interventions. However, they view these actions as addressing both the physical and existential needs of the patient. They observe that interventions aimed at symptom relief often alleviate anxiety, contributing to a more stable emotional state. In such moments, ACs note that patients often place their full trust in their care, feeling they have no other option.

But I believe that the patient also feels.when you’re in a situation where it’s a matter of life and death.I feel that they sort of just surrender themselves as well. So, then we just do what we need to do (Int7/Part1)

Additionally, ACs believe that their calm presence can help prevent the escalation of a patient’s distress during episodes of breathlessness. They reinforce this through verbal reassurance and physical closeness, such as standing near the patient and making eye contact to foster a sense of connection. By continually providing information and explaining what’s happening, they allow the patient to regain a sense of control over the situation, which in turn they believe helps to reduce patients feelings of anxiety.

If you describe things to the patient.then you create some kind of control for the patient too because usually.they’ve handed themselves over to the ambulance care, lying on the stretcher strapped down and such (Int5/Part1)

A well-coordinated teamwork within the ambulance crew ACs believe further enhances the patient’s sense of security. By demonstrating confidence through calm, controlled behavior and clear communication, ACs reassure patients that the situation is under control.

Because that becomes a treatment in itself somehow, that the patient feels they’re in safe hands. If I’m stressed and anxious.then the patient becomes that way too (Int6/Part1)

ACs find that reassuring patients they will receive all necessary assistance for relief fosters a sense of security. They also recognize the psychological impact of treatment. Administering oxygen, for example, often reassures patients, especially those familiar with the procedure. Additionally, the steady presence of ACs offers essential support, helping to alleviate feelings of isolation and fear in patients.

Relieving and easing anxiety is a factor. You could use drugs, but it often helps to approach them calmly, use oxygen. maybe hold their hand.things like that (Int4/Part1)

## Balancing patient autonomy with beneficence

ACs observe that patients with cardiovascular issues seek help quickly for breathlessness, while those with chronic lung diseases often delay. From the ACs’ point of view, several patients could avoid needing ambulance care if they sought medical attention earlier. ACs suggest this delay might be due to patients not wanting to burden ambulance services, a behavior most common among the elderly.

Yes, one shouldn’t seek unnecessarily.one shouldn’t cause trouble.one shouldn’t take someone else’s place, so to speak, and so on. Of course, there are exceptions as well. But generally, that’s how it is. They say it (Int1/Part1)

ACs note that especially patients with chronic pulmonary issues often delay seeking help to avoid hospital care, preferring on-site treatment at home. However, ACs find this group challenging because the underlying problem often lacks an easy solution, and patients are aware of this. When ACs arrive on site, they often find that patients have exhausted other options and feel compelled to seek help from ambulance services. ACs recognize that this situation can have serious consequences for the patients.

And then they start showing signs of ischemia and other issues on the ECG because they’ve struggled for so long. Yes, they are completely exhausted.and in very critical state (Int1/Part1)

ACs express frustration when patients refuse further medical assistance despite severe conditions. Some patients insist on staying home after initial treatments, believing they know best. This creates a conflict between ACs’ medical expertise and patients’ perceptions of their needs. ACs feel inadequate when leaving patients who may struggle at home but acknowledge that the ultimate decision rests with the patients. This dynamic presents challenges as ACs strive to ensure patients understand the risks while respecting their autonomy.

In those situations, I can sometimes feel a sense of helplessness when I leave the patient, like, I wanted this patient to. because maybe they can’t manage this at home (Int7/ Part2)

While ACs strive to offer information and recommendations to help patients make informed choices, they are also aware of the potential for increased workload. If they are called back to the same patients shortly after, it creates unnecessary stress, adding to the challenges of an already demanding role.

Often, I make this recommendation because I don’t want to come back in two hours to do the same thing.or have another colleague deal with it. It becomes unnecessary. // Our medical knowledge is then in conflict with the patient’s perception or preference (Int5/Part1)

ACs describe the movement of patients who rely on chronic oxygen therapy as a challenging task. Some patients accept temporarily disconnecting from their oxygen, while others refuse due to physical discomfort or psychological reasons. While ACs emphasize the importance of respecting patient choices, their limited knowledge of each patient’s comorbidities makes it challenging to balance patient autonomy with medical expertise.

But there are almost no easy patients. it’s challenging. To be honest, it’s tough for an AC who encounters this patient. We don’t know much about their background, maybe just a small part of it. We come into their lives and it’s not easy to just apply solutions in those situations. It’s difficult (Int2/Part1)

When ACs need to prioritize life-saving interventions over patient autonomy, moral dilemmas arise, especially when patients delay or resist care. This situation places ACs in the difficult position of balancing their professional responsibility with personal frustration. They are aware that overriding patient preferences in these moments may undermine trust and risk distancing the patient from them. At the same time, ACs emphasize that safeguarding the patient’s safety and chances of survival must take precedence, even if it comes at the expense of the patient’s immediate sense of trust and control.

And in some situations, you just have to.if we’re going to save this patient, we have to do this now and not later, but now. we have to. And sometimes, you might need to force a little. You have to think about why you’re doing this. Yes, I’m doing it for the patient. And then I can defend myself to myself, so to speak (Int1/Part1)

### Navigating relatives and their involvement

When caring for patients with breathlessness, ACs emphasize the importance of involving relatives, as their input can provide valuable insights, even though their level of engagement varies. ACs navigate potential conflicts, such as when relatives interfere and insist on hospitalization, while also addressing emotional concerns. They focus on building alignment with relatives by acknowledging their feelings, offering reassurance, and fostering trust and cooperation, especially in critical situations.

### Including relatives in care

ACs interact with relatives who can be both valuable resources and occasional challenges. Some relatives actively provide essential information, helping ACs understand the patient’s condition better and involve them in care. Others prefer to stay passive in the background, prompting ACs to adapt their approach. When relatives show little interest, ACs engage them less. The degree of relative involvement significantly influences their integration into the patient’s care, highlighting the importance of ACs being flexible and responsive.

But some are very involved and helpful.and then you include them in pretty much everything you do. ‘Now we’ve given this, and it will make breathing easier’ you know.like that (Int8/Part2)

In critical situations, ACs focus on life-saving actions, which often requires interrupting relatives and limiting their involvement during urgent care. Nevertheless, ACs consider that relatives can still provide vital information, such as contact details, allowing ACs to complete the patient’s medical profile later, when circumstances permit.

Okay, they’ve done something, they’ve left their phone number, and we’ll contact them when we can.’ Plus, it’s beneficial to get more information afterward because it’s not often possible to gather all the details in critical situations (Int7/Part1)

### Managing potential conflicts

ACs encounter situations where patients do not perceive a need for assistance. In such cases, they often experience that it is the relatives who interfere with the ACs’ decision-making and insist on hospitalization, even when the ACs assess that the patient could safely remain at home. To manage these situations, ACs prioritize addressing the relatives’ concerns while maintaining focus on delivering essential care, aiming to prevent unnecessary conflicts.

It can be quite challenging because the patient might express not wanting to go, but here are these three relatives insisting that they should. Even if we might think, ‘Well. this might not meet the criteria for admission’ .those three individuals are still there (Int7/Part1)

ACs sometimes face frustration from relatives who feel their loved ones have been treated unfairly, often due to concerns about care adequacy or dissatisfaction with previous hospital discharges. While ACs can’t change past events, they focus on providing present support. Managing relatives’ emotions while delivering care is challenging, but ACs strive to foster trust and collaboration by reassuring relatives of their commitment to providing the best possible care for breathless patients.

*It’s good to align with relatives. I often go with that approach.to align myself with the relative (Int5/Part1)*

### Responding to relative’s needs for support

ACs encounter situations where some relatives can be challenging to manage while caring for patients with breathlessness. At the same time, they recognize and appreciate the significant effort many relatives invest in supporting their loved ones. ACs emphasize the importance of involving relatives in the care process and acknowledging their concerns. They find it effective to listen to relatives and validate their feelings rather than question them, fostering a supportive and collaborative approach.

Many relatives do an incredible job with their loved ones.and that’s something to respect. You want to help alleviate the burden in any way you can (Int5/Part2)

In critical situations, ACs try to calm relatives by clearly showing that they are there to help as quickly and effectively as possible. They acknowledge the stress relatives often feel in these situations and address these concerns by requesting contact information to provide updates later. They believe this approach helps to alleviate some of the anxiety experienced by relatives during such moments.

This can be very tough for relatives in these situations. But it’s also about quickly noting down the phone number, ‘so I can contact you and update you once we’ve admitted the patient’ (Int7/Part1)

### Navigating one’s own capability in care

ACs face various challenges in providing care for breathless patients. Their approach is multifaceted, prioritizing stabilization and adapting methods based on the patient’s distress level. Building trust, conducting thorough interviews when possible, and combining clinical judgment with structured assessments are key. Clinical experience boosts their confidence, but they are aware of the emotional burden, especially when they can’t alleviate the patient’s anxiety. Effective teamwork is crucial for support and shared decision-making. ACs stives to manage stress and to maintain professionalism despite emotional demands. Their clinical competence and ability to handle unpredictability are essential to providing effective care, underscoring the importance of their own capabilities in care.

### To identify the primary cause

ACs use a multi-faceted approach to assess patients with breathlessness, adjusting their methods based on the severity of the condition. Initially, their primary focus is on regaining control over the patient’s breathing, with the patient interview taking a secondary role. Stabilizing the patient’s condition and addressing immediate symptoms is the priority. They understand that patients in severe distress often cannot communicate or are too focused on their breathing to respond to questions.

It’s difficult to get a full medical history. They’re more interested in just getting air (Int3/Part1)

Building trust with patients is vital for ACs during assessments. They adapt their interview techniques based on the patient’s condition, ensuring their approach is sensitive to the patient’s needs and distress level. ACs recognize that a patient’s story can provide key insights, helping identify contributing factors to the condition. This relationship streamlines the assessment process, leading to more effective care and quicker handovers to other professionals.

And then it’s the detective work we’re engaged in. We need to establish a certain relationship with patients for them to open up and tell us what happened. I mean, there are many aspects of a patient’s life that we need to consider when making our decisions, and perhaps not everyone wants to talk about them right away when we first arrive (Int5/Part2)

ACs note that, although they are generally able to identify the underlying cause of breathlessness, certain cases remain particularly challenging. These cases often involve multiple symptoms and signs, such as pain combined with several abnormal vital parameters, especially in patients with accumulated comorbidities. Some ACs feel comfortable with the limitations of a field diagnosis, while others experience a sense of powerlessness when no clear cause can be identified.

The patients are quite complex.everything is all over the place.vital signs. medical history. and just.it’s hard to piece together. No, it becomes frustrating (Int8/Part2)

A structured approach combined with clinical judgment is essential during assessments, according to ACs. While they acknowledge the critical role of vital signs in ensuring patient safety, they also emphasize the importance of non-verbal cues in the evaluation process. By integrating observations of non-verbal signs alongside standard measurements, ACs demonstrate how they use both clinical expertise and intuition to provide comprehensive care.

I mean.the patient’s body language, facial expressions, eyes.it’s really about using one’s clinical gaze, not always relying on vital signs (Int7/Part2)

ACs find that the cause of a patient’s distress is not always clear, making their judgment crucial in choosing which hospital is best equipped to address the patient’s primary complaint. They emphasize a flexible, open-minded approach during assessments, even with prior patient information. This adaptability is essential for managing the multifactorial nature of breathlessness, which often involves overlapping symptoms. While ruling out severe conditions is a priority, ACs remain cautious about focusing too narrowly on a single diagnosis to avoid errors or overlooking critical information.

So, it’s good that you took that saturation check, right? It’s easy to be misled.because your clinical judgment isn’t 100% (Int5/Part1)

### The impact of knowledge and clinical experience

ACs describe how professional knowledge and accumulated clinical experience strengthen their self-confidence and ability to provide safe care for breathless patients. While they generally succeed in alleviating distress, rare or complex cases are perceived as particularly challenging. Such situations are especially demanding when clinical experience is limited, a view also emphasized by experienced ACs, who stress that uncertainty and vulnerability are inevitable when new to the profession. In such encounters, some ACs rely heavily on protocols and structured assessments, which provide essential guidance but may prolong decision-making and limit the ability to address patients’ existential needs.

You feel a bit of pressure that you have to solve this. I mean, I am this person’s lifeline here. She’s asking me to save her from dying, and of course, I want to do that. So, yes, I feel the pressure to do something. But you also have a limited number of tools in your own toolbox (Int8/Part1)

Feelings of isolation arise when ACs lack support in making critical decisions, particularly when working with equally inexperienced colleagues. In these situations, they describe carrying the main responsibility for complex assessments while being unsure how to prioritize or where to begin. This sense of being professionally alone is reinforced when protocols provide structure but do not address patients’ existential needs, leaving ACs vulnerable in their caring role. Despite these challenges, ACs stress the importance of giving less experienced colleagues the opportunity to gradually build confidence and competence, recognizing professional growth as a continuous and essential process.

And the longer you’ve worked, the easier I think it becomes to maintain some objectivity. When you’re very new to the job, you often go ‘all in’ with the patient and might miss the bigger picture around them (Int5/Part2)

ACs experience that pairing newer colleagues with more seasoned ones allows the less experienced to observe decision-making processes and gradually build confidence in handling complex cases beyond protocol-based responses. It also lightens their sense of responsibility, enabling them to focus more confidently on patient care, knowing they have support when challenges arise.

So, ideally you ride with someone who has been around for a while. or at least a bit longer. someone you can rely on for support (Int1/Part1)

Some ACs describe how they often take flexible actions beyond standard guidelines to create a calming environment for patients. They emphasize that such actions lie within their clinical competence and knowledge to perform. However, ACs sometimes experience that their expertise is not fully recognized, especially when contacting on-call junior doctors remotely for support. This can lead to frustration, particularly when they are not given the opportunity to engage in open discussions or share their insights.

It can be very frustrating when we can’t discuss the situation or provide input, especially when their decisions might not fully align with what we believe is necessary based on our experience (Int1/Part1)

Moreover, ACs emphasize the importance of regularly encountering breathless patients to maintain and update their clinical skills. They describe how prior clinical experience enhances their ability to manage complex cases effectively in ambulance care. Simulation training is also occasionally used at workplaces, primarily to support interprofessional collaboration or practice specific skills such as cardiopulmonary resuscitation. However, it is noted that access to simulation activities, including those beyond resuscitation, varies between workplaces. These experiences enhance ACs’ confidence, providing trust in their clinical judgment, security in rapid decision-making, and assurance when performing interventions. Familiarity with respiratory distress enables them to approach these situations with calmness and composure, even under pressure.

I believe it’s beneficial to have a few years of clinical experience when entering ambulance care. We’re two ambulance clinicians trying to handle whatever comes our way, and it’s not always simple. These patients with breathlessness don’t seem particularly challenging to me, though (Int2/Part1)

Despite having extensive clinical experience within ambulance services, some ACs still face challenges, especially when patients exhibit death anxiety, which can be difficult to address. Despite their expertise, some ACs acknowledge that their self-confidence may fluctuate despite broad knowledge. They view this uncertainty positively, as it helps keep them grounded and prevents overconfidence.

Self-confidence fluctuates somewhat, and I think it’s healthy to have that. to not take yourself for granted and believe you can handle everything (Int7/Part1)

### The value of teamwork

ACs believe that effective teamwork is crucial for providing safe care to patients experiencing breathlessness. They describe mutual trust and the ability to rely on each other as essential, particularly in high-pressure situations. Communication through nonverbal cues, such as body language and brief eye contact, allows them to collaborate effectively even under stress. ACs also note that they can offer subtle support to a colleague when they recognize uncertainty or the need for guidance in managing a patient’s respiratory distress.

Yes, but then I’ll step in and take over. Or not take over, but maybe offer some support. ‘Hey, have you considered this? Do you want to administer an inhalation now?’ Just like that. You have to do it a bit subtly (Int1/Part1)

When working as a team, ACs feel secure with a colleague by their side, which helps alleviate emotional burdens and share responsibilities in addressing a patient’s breathing difficulties. Some ACs find comfort in quickly assessing the patient’s condition together, enhancing decision-making and providing emotional reassurance. Nonverbal communication also streamlines their response in urgent situations, allowing them to focus on the patient’s needs without distraction.

Now my colleague can sit with the patient, so we take turns. I didn’t want it to become apparent that I was unsure of what to do next. So, we switched places, not because she could do more than I could, but to manage the situation better together ,(Int6/Part1)

Some ACs emphasize the challenges of managing patient care independently. They describe how the lack of immediate support increases their emotional strain, particularly when attending to breathless patients in high-stress situations. Without a colleague to lean on, maintaining calm and focus becomes more difficult. In such scenarios, ACs must rely entirely on their own skills and coping mechanisms to meet the demands of patient care.

I face challenges when I’m operating as a single responder because I don’t have anyone to help clear my mental blocks. In those cases, I need to rely on my own tools (Int1/Part1)

### Managing stress and futility

When patients remain stable despite breathlessness, ACs stay calm and work effortlessly. However, if a patient’s condition worsens, some ACs remain unaffected by the urgency, while others feel increased pressure to “solve” the situation. This highlights the emotional demands ACs face as they balance professional responsibility with empathy in their care. Despite field limitations, all ACs remain dedicated to providing the best care possible, demonstrating their commitment to meeting each patient’s needs.

It’s really disappointing when you can’t reach someone who’s very ill, and you fail to alleviate their anxiety. It’s frustrating if you end up rushing them or if other issues arise, as you genuinely want to help the person (Int4/Part2)

When caring for critically ill patients, ACs feel a heightened responsibility to act quickly and effectively, aware of the potential for rapid deterioration. This becomes particularly challenging when patients worsen despite adherence to standard protocols and interventions, creating situations in which ACs must navigate the limits of what can be achieved. Some ACs consciously try to manage these situations of potential futility by focusing on what they *can* influence, while others feel vulnerable, experiencing self-doubt and questioning their adequacy.

She sat there screaming for 30 min from anxiety, and you don’t know what to do. It’s really tough. You feel like you’ve done everything you can, but it’s still. yeah, it’s quite tough actually (Int8/Part1)

ACs emphasize that stress when caring for patients with breathlessness can affect their objectivity in assessing the patient’s condition and making clinical decisions. In some cases, patients’ behaviors, such as panic or expressions of death anxiety, may trigger emotional reactions in ACs, which can challenge their ability to convey confidence and provide reassurance towards patients. To minimize the risk of increasing patient distress, ACs actively strive to regulate their own emotional responses throughout the care encounter.

I don’t want my anxiety to be visible. I try to manage my own thoughts, even if I’m thinking ‘This might not end well’. I don’t want the patient to sense my concern, as it might increase their own anxiety. So I make an effort to project confidence and professionalism, even if I personally have doubts about the outcome. It’s about maintaining a calm and reassuring presence (Int6/Part1)

To manage the physical and existential demands of patient care, ACs emphasize the importance of maintaining professionalism and managing their own anxiety. Their coping strategies reassure patients and help them address feelings of helplessness. On the other hand, ACs also stress that unpredictability is a constant in their work, requiring effective stress management. They acknowledge that, despite the discomfort of some situations, they chose this profession knowing it involves confronting unsettling experiences.

Sure, one might perceive things to be more or less comfortable or feel.But I’ve chosen this job, and I’ll witness things that no other person will ever see. That’s what I’m prepared to do (Int2/Part1)

## Discussion

This study provides important insights into ACs experiences of caring for patients with breathlessness, an area that has so far received limited attention in pre-hospital research. By addressing this gap, the study makes a unique contribution to understanding how ACs manage both the clinical and emotional challenges associated with caring for these patients. The main finding underscores the importance in care, of balancing medical interventions with a calm and professional demeanor. ACs emphasized that such balance is essential for helping patients regain a sense of control in moments of severe anxiety, which they also perceive is central to fostering trust. However, this was especially challenging in suspected critical cases with multiple symptoms, abnormal vitals, and high uncertainty while also addressing existential needs. From the patients’ perspective, previous research on those suffering from breathlessness demonstrates patients’ feelings of anxiety and loss of control [24, 43], and a critical need for empathetic, calm guidance from clinicians. This includes recognizing the multifaceted nature of breathlessness and addressing both physical and existential needs [30, 44]. The ACs in our study indicated that previous clinical experience plays an important role in managing such complex situations. This finding suggests that accumulated clinical experience may contribute to greater confidence and a more nuanced approach to both the medical and emotional aspects of care. However, it is reasonable to assume, and this aligns with previous research, that challenges are most evident early in a professional career, when clinical experience is limited and clinical judgment is still developing [45]. More experienced clinicians are generally better equipped to handle these challenges, enabling a more holistic approach to patient care [46]. Our results further imply that ACs can broaden their focus to encompass wider aspects of patient care once they feel confident and secure in performing medical assessments. A lack of such confidence, combined with exposure to high levels of stress and uncertainty in care provision, may contribute to cognitive load. *Cognitive load* refers to the mental effort needed to process information and make decisions under complex or uncertain conditions [47]. In the pre-hospital setting, this cognitive load can cause ACs to narrow their focus to immediate medical tasks. As a result, they may overlook other vital aspects of patient care, such as addressing existential concerns or maintaining effective communication [48]. In addition to cognitive demands, our findings indicate that the emotional dimension of care may represent another challenge for ACs. Some described how the intensity of patients’ emotional distress could transfer to themselves, making it difficult to maintain the calm presence their patients required. This highlights the dual challenge of simultaneously providing reassurance and managing one’s own emotional responses, as clinicians’ difficulties in regulating their emotions can influence both decision-making and the quality of care [49, 50]. The emotional challenges described by ACs also highlight an important ethical aspect of care. When ACs struggle to manage the patient’s emotional distress, they face the tension between staying calm and professional while also responding compassionately to the patient’s suffering. From a caring science perspective, these situations show that care is relational, as ACs are affected by the patient’s vulnerability while at the same time carrying a responsibility to support and relieve suffering. Emotional awareness is therefore not separate from clinical competence but forms a central part of ethical caring [51, 52].

Moreover, stress and anxiety among ACs are known to impair judgment, increasing the risk of errors and misunderstandings that may compromise patient safety, particularly in critical situations requiring rapid and accurate assessments [53, 54]. It does not only risk impairing clinical judgment but can also limit the ACs capacity to engage in the relational and supportive aspects of care that are fundamental to alleviating patient suffering. When cognitive resources are heavily taxed by complex decision-making, uncertainty, or acute stressors, the ACs ability to be fully present, attuned, and responsive to the patient’s psychological and existential needs may be compromised [55]. According to patients experiencing breathlessness, encountering such a presence is vital for fostering feelings of safety and trust, which in turn help them cope with fear, loss of control, and existential distress [30]. Elevated cognitive load in ACs may narrow the caring space, potentially shifting the focus from holistic care to task-oriented actions, even in situations where patients are in great need of emotional support. These insights underscore the need for interventions that strengthen emotional resilience and provide organizational support to promote both patient safety and ACs well-being.

Being paired with more experienced colleagues was desired by ACs in our results, who viewed mentorship as an effective way to reduce stress, enhance care quality, and foster professional competence. Importantly, the development of competencies must be ongoing, especially to meet the demands of rare respiratory cases, since confidence grounded in clinical expertise and solid medical knowledge enables healthcare providers to focus beyond immediate interventions [56]. Additionally, knowledge of human physiology and pathophysiology is fundamental for medical decision-making, as it helps ACs understand disease mechanisms, predict patient responses, and make informed decisions. Thereby reducing the risk of cognitive load [57]. Moreover, personal confidence is also described being influenced by factors such as organizational support and collegial trust. Strengthening these factors is a way to enhance both performance and confidence among ACs in patient assessments [58].

Previous research indicates that the way ACs perceive their own competence is closely linked to patient outcomes [59], even here highlighting the importance of ongoing education and clinical training to enhance their professional skills and readiness. Structured training programs that integrate theoretical knowledge, practical skills, and strategies for managing stress and emotional challenges are described as essential for addressing these gaps and fostering improved care delivery [60, 61], and ensuring patient safety [62]. Training programs that incorporate cognitive load management strategies, such as simulation-based- and stress exposure training, is therefore a way to equip ACs with the skills necessary to address immediate medical needs while also attending to broader aspects of patient care [63]. It also enables to build the confidence and coping mechanisms necessary for handling high-pressure scenarios effectively [64, 65]. Additionally, clinical exchange with in-hospital settings offers opportunities for ACs to observe and engage in the acute management of severe respiratory cases. Such exchanges are a way to help bridge gaps in clinical experience, enhance decision-making capacity, and complement the reflective practice central to professional development. Ultimately, by combining ongoing education, simulation training, and clinical exchange, ambulance services can better support both novice and experienced ACs in managing the complex clinical and emotional demands of caring for patients with breathlessness.

### Strengths and limitations

This study has several strengths. All authors are experienced pre-hospital researchers, with two in pre-hospital simulation research, which informed the study design. Two of the authors are RNs, one with specialist training in intensive care and the other in ambulance care, both with extensive clinical experience. The use of high-fidelity simulation provided participants with a clear introduction prior to interviews and was particularly valuable given limited direct access to the pre-hospital field [66]. Dyadic interviews facilitated richer insights by capturing shared experiences [67], and the involvement of multiple interviewers enhanced credibility through diverse perspectives [68]. The study presents a description of participants with varied backgrounds, clinical experiences, and ambulance stations, illustrating diverse cases. In addition to the main themes, minor themes and less common perspectives are also discussed, providing a nuanced understanding of the data.

However, certain limitations should be acknowledged. Although snowball sampling was used, which can theoretically introduce selection bias, the data collection process ensured independence between participants. All simulated scenarios and interviews were conducted consecutively, leaving no opportunity for participants to discuss the scenarios or interviews with each other. This procedure minimized the risk that potential selection bias affected the findings. None of the interviewers had a professional background in ambulance care, which may have helped reduce assumptions and allowed participants to describe their experiences more freely. One interviewer had prior personal and professional contact with three participants. These interviews were conducted by another interviewer with no prior relationship to minimize potential influence. In some dyadic interviews, more experienced participants tended to dominate the discussion, potentially limiting input from less experienced colleagues [67]. Although interviewers sought to engage both participants, this dynamic may have influenced the findings. Data analysis primarily addressed manifest content. However, during categorization some degree of latent content analysis was unavoidable incorporated to achieve a comprehensive understanding, which is recognized as a strength [69]. This also introduced the risk of researcher preunderstanding shaping interpretation. Abstraction into categories may have led to a loss of certain nuances in participants’ experiences. Data were collected in 2021, and it is possible that clinical practices, system pressures, and resource availability have changed since then. This should be considered when interpreting the findings and their transferability to current pre-hospital care contexts. Another limitation concerns the RN-based Emergency medical service (EMS) model in Sweden. All ACs are registered nurses, although their specialist training varies, which may influence how they perceive and manage patients with breathlessness, particularly given the strong holistic orientation within nursing. This differs from international paramedic-led systems. It is important to acknowledge that paramedic education varies widely across the world, ranging from university-based programs leading to a bachelor’s degree to short, individual courses. While caring science is profession-neutral and applicable across EMS contexts, the nurse-led structure may shape how these principles are enacted in practice. Transferability should therefore be considered with caution. Finally, the study was conducted in a limited number of ambulance stations within a specific Swedish region. While the Swedish context and organization of ambulance services may affect transferability to other healthcare systems, the findings nevertheless offer important insights into ambulance care and the care of breathless patients, which may be applicable across healthcare systems at different levels internationally.

## Conclusion

While ACs generally understand what patients with breathlessness need upon arrival, it is the rare and complex cases that present the greatest challenges. These infrequent but critical situations highlight the importance of clinical experience in supporting effective decision-making and comprehensive patient assessment. Caring for breathless patients imposes substantial cognitive and emotional demands on ACs, requiring them to balance patient needs with managing their own stress. The findings emphasize that clinical experience fosters confidence and competence, enabling ACs to deliver high-quality care under pressure. Structured clinical exchange and regular simulation training are essential strategies to build and sustain these competencies, ultimately improving pre-hospital outcomes for patients with breathlessness. Pairing less experienced ACs with seasoned colleagues could improve care quality and support the development of professional competence.

## Electronic Supplementary Material

Below is the link to the electronic supplementary material.

Additional file 1

## Data Availability

The datasets used and analyzed during the current study are available from the corresponding author on reasonable request.

## Abbreviations

AC: Ambulance clinician
EMS: Emergency medical services
RN: Registered nurse
